# Obesity as a risk factor in atrial fibrillation and heart failure

**DOI:** 10.1007/s40200-023-01332-z

**Published:** 2023-10-25

**Authors:** Jakub Jurica, Martin Jozef Péč, Jakub Benko, Tomáš Bolek, Peter Galajda, Marián Mokáň, Matej Samoš

**Affiliations:** 1 Department of Internal Medicine I, Jessenius Faculty of Medicine in Martin, Comenius University in Bratislava, Kollarova 2, 036 59 Martin, Slovak Republic; 2 Division of Acute and Interventional Cardiology, Department of Cardiology and Angiology II, Mid-Slovakian Institute of Heart and Vessel Diseases (SÚSCCH) in Banská Bystrica, Banská Bystrica, Slovakia

**Keywords:** Atrial fibrillation, Heart failure, Obesity, Obesity paradox

## Abstract

**Objectives:**

The aim of this article is to provide an insight into the role of obesity as a risk factor, and as a potential etiologic agent of atrial fibrillation (AF) and heart failure (HF).

**Methods:**

A narrative (non-systematic) review article summarizing currently available data regarding the interaction between obesity, AF and HF.

**Results:**

Obesity is considered a risk factor of AF and chronic HF. Multiple recent studies indicate that obesity is also a potential causal factor in the development of AF and HF, the elucidation of pathological mechanisms of which could help devise new diagnostic and therapeutic modalities for these conditions. The discussion about obesity in relation to HF cannot omit the so-called obesity paradox, which represents a dilemma for clinicians, and it is still a source of irregularities regarding the strategy of weight reduction in obese patients with HF. Recently, the obesity paradox has also been assumed to play a role in the relationship between obesity and thromboembolic complications of AF.

**Conclusions:**

Obesity is an independent and modifiable risk factor for AF and HF. In addition, there is an increasing volume of experimental and clinical data that suggests an important role of the epicardial adipose tissue in the pathophysiology of AF. However, several issues, such as the issue of optimal pharmacotherapy and weight reduction strategy in obese patients with HF remains still unanswered, and open for future investigation.

## Introduction

Obesity is currently regarded as a worldwide pandemic with an estimated prevalence of one eighth of the world's population, 30% of the European population with a rising trend [1], having a detrimental impact on the population's health, healthcare systems as well as the economic systems around the world. Its complications give rise to increased morbidity and mortality, particularly due to accompanying cardiovascular diseases. Approximately 5 million people die from obesity each year [2]. Obesity is tightly linked to other diseases, such as type 2 diabetes (T2D) and heart failure (HF). In the light of new evidence it is necessary to discuss obesity as a factor that contributes to the development of HF. HF represents a leading cause of morbidity and mortality worldwide.

In addition, atrial fibrillation (AF) is the most common clinically significant arrhythmia in adult population, and it might lead to possibly fatal complications in the form of cardioembolic stroke. The prospect of obesity being a risk factor for AF is subject to an ongoing debate that has only intensified recently since it was demonstrated that an increase in the Body Mass Index (BMI) is associated with a significant risk of the development of AF.

The so-called obesity paradox as described in the literature is an inseparable part of the discussion about obesity and the increased risk of cardiovascular disease. It is defined by better prognosis and lower mortality in patients, who are overweight or Class 1 obese with cardiovascular disease, especially HF, compared to patients with normal weight. This paradox represents a therapeutic dilemma within the context of weight reduction strategies as there are no clearly established recommendations for targets in the management of weight reduction as part of HF therapy. The obesity paradox does not seem to be limited to just HF but it is also reflected in various other clinical entities including AF, where although a greater BMI is related to an increased risk of AF incidence, there is an inverse relationship between the BMI and the degree of severity of stroke in patients with AF.

The aim of this article is to provide a complex review of currently available data regarding the link between obesity, HF and AF; and about the impact of obesity on clinical course, therapy and prognosis of these diseases.

## Obesity and AF

Obesity is a chronic metabolic disease characterised by excessive and abnormal accumulation of adipose tissue in the body. There is a number of methods available to measure and evaluate obesity with the BMI being the most frequently used one and expressed in the unit of kg/m^2^. The World Health Organisation defines the various degrees of obesity based on the BMI value: overweight 25.0 – 29.9 kg/m^2^, Class 1 obesity 30.0 – 34.9 kg/m^2^, Class 2 obesity 35.0 – 39.9 kg/m^2^, Class 3 obesity 40.0 – 49.9 kg/m^2^ and severe morbid obesity with the BMI over 50.0 kg/m^2^ [3]. However, there are certain limitations to this method, as it cannot interpret the distribution of fat in the body accurately, neither does it take into account the body composition including muscle mass. The measurement of waist circumference is able to evaluate central (abdominal) obesity and it is therefore a more suitable method to assess obesity in clinical practice [4]. The aforementioned is supported by the fact that this method is used as one of the criteria of metabolic syndrome and it emphasizes that predominantely visceral adipose tissue and its distribution, rather than the total body fat, play a key role in the development of cardiovascular disease [2]. Obesity exerts its effects on the human organism in the form of mechanical complications caused by the adipose tissue itself, synthesis of various metabolic products that result in metabolic complications associated with obesity, and other, not entirely elucidated mechanisms to date, which together increase the mortality of obese individuals [4].

### Obesity and AF risk

Obesity is currently considered a modifiable risk factor not only for the incidence, but also for the progression and recurrence of AF [5]. Moreover, it is the second most attributable risk factor for AF after arterial hypertension [6]. Obesity was associated with a new-onset AF independent of age, T2D, arterial hypertension and gender in a recent study performed by Foy et al. on an American cohort of 67 238 patients [7]. The Framingham study showed that at BMI > 25 kg/m^2^, the risk of AF increased by 4% for every 1 kg/m^2^ rise in the BMI [8], which was further supported by an extensive metaanalysis of 626 603 individuals, in which every 5 kg/m^2^ increase in the BMI corresponded to a 29% rise in the incidence of AF [9].

Obesity seems to be the key trigger of the worldwide exponential growth in cases of AF as just in Europe alone the number of estimated cases is expected to grow from 8.8 million in 2010 to roughly 17.9 million by 2060 [10]. The relationship between obesity and AF is a complex one, the AF risk is rather attributed to cardiovascular risk factors accompanying obesity as for example T2D, arterial hypertension and others mentioned above. However, in another study involving 400 000 patients, who had a high BMI, but were “metabolically healthy“, i.e. without severe comorbidities, it was demonstrated that “metabolically healthy obese individuals” also had an increased risk of developing AF compared to healthy non-obese ones [11]. This finding together with the results from a previously mentioned study performed by Foy et al. [7] indicate that the relationship between obesity and AF is independent of any concomitant comorbidities. The apparent obesity paradox is present here as well as in other cardiovascular conditions, particularly in association with the mortality of overweight and Class 1 obese patients with AF, who show a lower mortality in comparison with nonobese patients. There is even an inverse association between the BMI and the degree of severity of stroke in patients with acute ischaemic stroke and non-valvular AF [12, 13]*.*

**Fig. 1:**
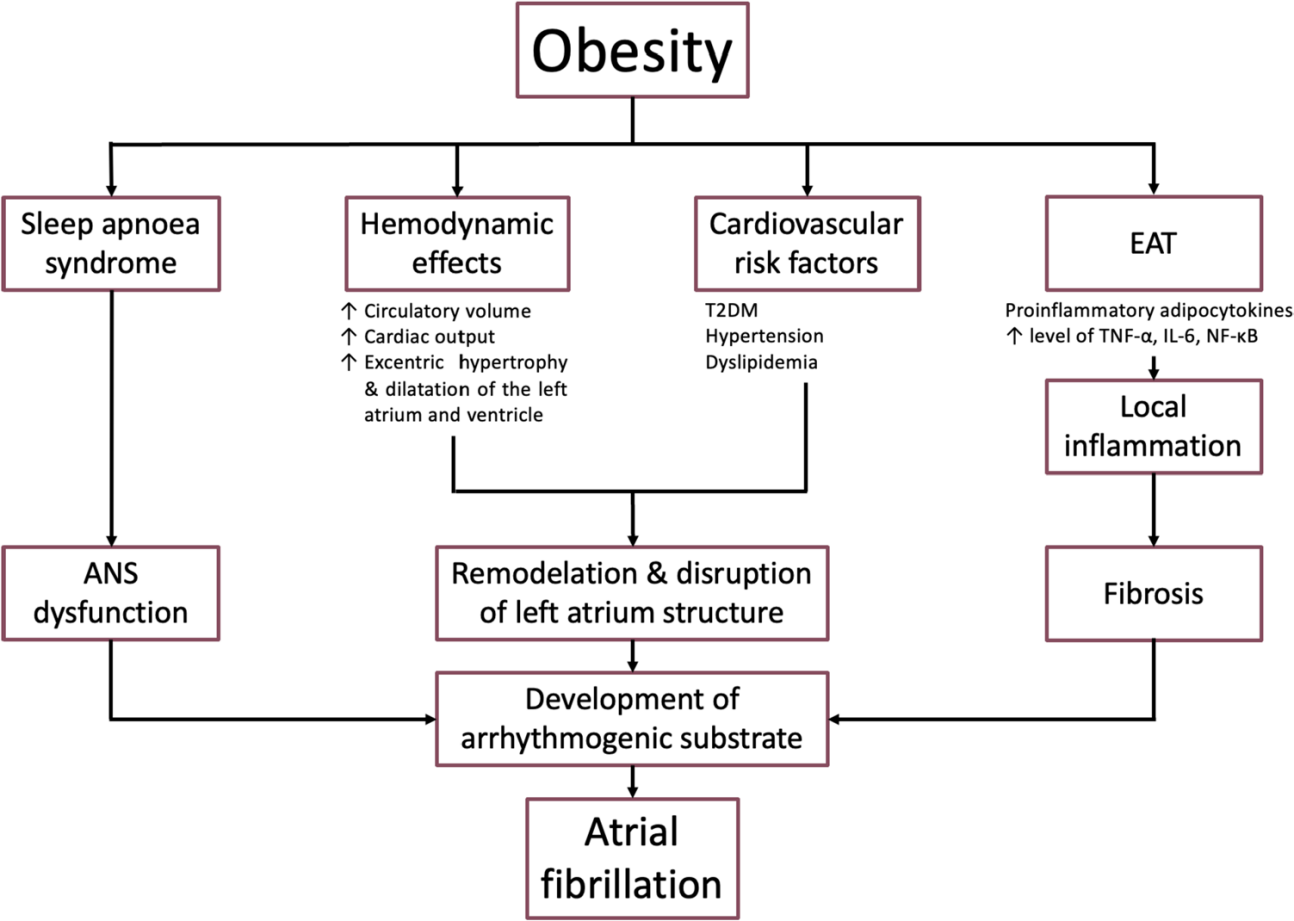
Link between obesity and atrial fibrillation (ANS – autonomic nervous system; EAT – epicardial adipose tissue; IL-6 – interleukin 6; NF-κΒ – nuclear factor kappa B; T2DM – type 2 diabetes mellitus; TNF-α – tumor necrosis factor alpha)

The pathomechanisms involved in the development of AF in obese patients are diverse and subject to an ongoing and extensive research (Fig. 1). The hemodynamic effects of obesity are associated with an increased total circulatory volume, which requires an elevation in the cardiac output. A volume overload of the left heart develops over time alongside excentric or concentric hypertrophy and dilatation of the left ventricle and left atrium as a result of increased filling pressures. This leads to structural abnormalities of the heart chambers, remodelation and disruption of the left atrial architecture, which in combination with the syndrome of obstructive sleep apnoea, frequently associated with obesity and altering the tension of autonomic nervous system (ANS), represents the ideal substrate for the formation and persistence of AF [14]*.* Local inflammation plays an important role in the obesity-AF relationship. Epicardial adipose tissue (EAT) is known to produce various proinflammatory adipocytokines and growth factors, which diffuse to the myocardium due to the identical blood supply. This claim is supported by research studies of patients with AF undergoing valvular surgery, where high blood levels of nuclear factor kappa beta (NF-κΒ), tumor necrosis factor-alpha (TNF-α) and inteleukin 6 (IL-6) as well as increased fibrosis and lympho-monocytic infiltration were observed [15]. Fibrosis is actually a pivotal factor in the development of an arrhythmogenic substrate while proinflammatory factors with paracrine activity such as activin A and matrix metalloproteinases synthesised by the EAT participate in fibrogenesis [16]. The impact of direct myocardial infiltration with fat and subsequent lipotoxicity also contributes to structural and electrophysiological modifications of the atria. In addition, the ANS dysfunction secondary to obesity (with concurrent sleep apnoea) was shown to be a trigger for AF in animal models. Taking into account the fact that nerve plexuses of the ANS are localised in the vicinity of the EAT within the epicardium, the paracrine effect of the mediators produced by the EAT can potentiate the development of AF through this mechanism [17]. Finally, the data from the observational studies indicate that the volume of the EAT correlates with an increased risk of AF [18]*.* In summary, the obesity-related atrial remodelling seems to be complex, and there is solid evidence that obesity might directly contribute to development and persistence of AF.

### Obesity and pharmacological therapy of AF

The effective anticoagulation is the basis of the pharmacological prevention of stroke or systemic embolism in AF [19]. At present, the group of direct oral anticoagulants (DOAC) is the preferred choice over vitamin K antagonists (VKA). Hemodynamically significant mitral stenosis and the presence of a prosthetic heart valve represent the contraindications of the DOAC therapy in AF. With respect to anticoagulation therapy of obese patients with the BMI > 40 kg/m^2^ or the weight over 120 kg, the International society for hemostasis and thrombosis does not recommend DOAC in this group of patients due to a lack of clinical data about efficacy and safety [19]. Warfarin remains the drug of choice in this group, although recent studies stressed the need for increased dosage in patients with the BMI > 40 kg/m^2^, which risks exceeding the therapeutic range of warfarin, not to mention other complications that arise from the great variabilitity of the effect and interactions of this coumarin derivative [20]. This group of patients may, therefore, benefit from DOAC; however, futher research is required in this field. For example, the study performed by Kapla et al. evaluating obese patients including a subgroup of morbidly obese treated with warfarin or DOAC undergoing electrical cardioversion (ECV) for AF showed a very low incidence of stroke (with no stroke in the subgroup with the BMI > 40 kg/m^2^) one month after the procedure [21]. This approach is supported by the recent metanalysis comparing the efficacy and safety of the DOAC in obese and nonobese patients with AF, which demonstrated that obese patients taking t DOAC had a lower risk of stroke/systemic embolism and a similar risk of bleeding compared to the nonobese ones [22].

As mentioned above, the EAT participates in the development and progression of AF, which is not only determined by the positive correlation between the EAT volume and the AF risk, but also by the documented presence of proinflammatory mediators produced by this tissue in the myocardium of the patients with AF. Logically, the EAT and its possible therapeutic modification have recently become the subject of research as another modality of treatment besides the well-known strategies of rate control and rhythm control. The disruption and termination of the EAT inflammatory cascade using specific antibodies against adipocytokines or profibrotic agents can diminish the EAT activity and prevent new-onset AF [23]. Drugs, which can possibly reduce the EAT volume are currently studied. Glucagon-like peptide 1 (GLP-1) analogues, namely liraglutid, can serve as an example with a demonstrated 40% reduction in the volume of EAT on echocardiography in a cohort of diabetic patients [24]. This effect may be explained by the well-known antiobesitic activity of incretins through induction of satiety, inhibiton of gastric emptying, stimulation of insulin secretion and reduction of postprandial glycaemia. Sodium-glucose co-transporter 2inhibitors (SGLT2i) are another group of anti-diabetic drugs that are being researched as a means of therapy as well as primary prevention of AF in obese patients. Dapagliflozin, for instance, has a well-documented effect on weight reduction and reduction of EAT volume as well. Furthermore, it has been shown to have anti-inflammatory activity by decreasing the TNF-α serum levels in diabetics [25]. However, further studies are required to evaluate the effect of these drugs in reducing the AF risk in obese patients.

### Obesity and non-pharmacological therapy of AF

Risk factor control and lifestyle choices are in spotlight as of late and they are being debated as a potential fourth pillar of the AF therapy alongside the anticoagulation therapy, rate control and rhythm control strategies [26]. Control of modifiable risk factors is the foundation of non-pharmacological therapy of AF. Hence, the goal is to maintain good compensation of T2D along with weight reduction, control of blood pressure, reduction of serum levels of lipids, smoking cessation, limiting alchohol consumption and improving physical endurance (so called cardiorespiratory fitness). This strategy is supported by the demonstrated reverse cardiac remodelation and regression of AF from a persistent to a paroxysmal form or its complete disappearance [27]. Furthermore, cardiorespiratory fitness is being currently studied as a significant factor in the reduction of AF. This observation was described in several studies. For example, Qureshi et al. documented a 7% lower risk of the incidence of AF for every one metabolic equivalent gained during treadmill exercise [28]. This lower risk is accompanied by improved survival and decreased burden of arrhythmia in terms of symptom reduction [27]. Dietary measures or bariatric surgery are other options to achieve a reduction in body weight. While diet and caloric deficit are not supported by adequate data from clinical trials focused on the AF risk reduction, bariatric surgery may be able to achieve significant and sustainable weight loss and subsequent reduction in the AF risk [28]. This was documented in the Swedish SOS (The Swedish obese subjects) study, where 2000 patients undergoing bariatric surgery were followed over a period of 19 years and there was a 29% reduction in the risk of new-onset AF in comparison with a control group formed by 2021 obese individuals [29]. In addition, a retrospective study with 239 morbidly obese patients undergoing ablation for AF demonstrated a lower recurrence of AF in those patients that had undergone bariatric surgery prior to ablation [30].

In terms of the rhythm control strategy, ECV or catheter radiofrequency ablation (CRFA) can be considered alongside the pharmacological therapy of AF. Each of these methods has its own indications, clinical justification and its efficacy is associated with factors like age, symptomatology, absence/presence of organic heart disease etc. However, it was shown that ECV had a lower success rate in patients with a greater body weight [31]. Perhaps that can be explained by a decreased amount of energy reaching and affecting the atria in patients with a greater body weight and therefore leading to ineffective depolarisation without establishing the sinus rhythm. This theory is supported by another study where utilising a greater amount of energy was associated with a higher probability of ECV success [32]. Additionally, a recent prospective study examining the efficacy and safety of AF cryoballoon ablation showed no differences in procedural outcomes between obese (251 patients) and non-obese individuals (698) during a median follow-up of 15 months [33]. However, right now, it is not entirely clear how obesity affects the outcomes of catheter ablation of AF. Although early studies with CRFA in obese individuals reported that CRFA was effective in obese ones, with consistent improvement in quality of life across all BMI categories [34], other studies did not show clearly promising results. In these prospective studies, obesity (or only extreme obesity) was significantly associated with long-term AF recurrence after CRFA [35], procedural failure [36], low procedural success rate especially in those with non-paroxysmal forms (extreme obesity) [37], increased risk for long-term redo AF interventions [38], and higher rate of procedure-related complications (especially in morbidly obese individuals) [39]. Going further, Tabaja et al. [40] recently reported that CRFA for AF was safe with a low risk of procedural complications (1.5%) and lead to improvement in quality of life regardless of BMI. In this study, only extreme obesity (defined as BMI ≥ 40 kg/m2) was associated with reduced CRFA success. Finally, there are limited data on efficacy and safety of novel catheter ablation techniques (cryoballoon ablation, pulsed field ablation) in obese individuals, as studies with these novel techniques had generally excluded patients with morbid obesity, and did not report sub-analysis of their results in obese patients. As mentioned, for cryoballoon ablation, one study is available (with no difference in procedural outcomes regarding to presence of obesity) [33]; unfortunately, there is no study reporting efficacy/safety data for pulsed field ablation procedures. These novel techniques might improve the outcomes of AF ablation procedures in obese patients. Definitely, future studies on this issue are warranted.

Summarizing, these data suggest that early rhythm control strategy could be preffered in obese AF patients to reduce the risk of AF-related complications; however, it is not clear what strategy for rhythm control should be preferred, as studies suggest higher risk of procedural failure in (morbidly) obese individuals. Undoubtedly, further research in this area is definitely needed for any final recommendations.

## Obesity and HF

The syndrome of HF is the leading cause of morbidity and mortality with a 2-3% prevalence in the population of the developed countries with a rising trend [41]. In spite of the improved survival rate, the mortality of HF still remains a serious issue, as roughly 50% of patients die within five years of establishing the diagnosis of HF [42].

Obesity has been described as an independent risk factor in the development of HF [43]. The risk of developing HF is two times greater in patients with the BMI > 30 kg/m^2^ in comparison with healthy, non-obese individuals [44]. In addition, the results of epidemiological studies point out a causal relationship between obesity and the development of HF, because the HF risk increases by 5% in females and by 7% in males for every 1kg/m^2^ gain in the BMI [45]. Obesity can cause HF either by inducing its own hemodynamic and myocardial changes also known as obesity cardiomyopathy, and/or by predisposing to other HF risk factors such as T2D, arterial hypertension, dyslipoproteinemia, atherosclerosis etc. Obesity cardiomyopathy could be defined as a state, in which patients develop cardiac dysfunction that cannot be attributed to other conventional HF causes [46]. This cardiomyopathy is estimated to be the cause of 11% HF cases in males and 14% HF cases in females [47]. The pathomechanisms participating in the obesity-HF relationship are diverse. Hemodynamically, as mentioned above, there is an increased cardiac output and venous return with the dilatation of the ventricles and an increase in systemic vascular resistance. Moreover, long-lasting obesity is associated with hypertrophy and dilatation of the left ventricle, which is highlighted by the positive correlation between the BMI and the size of the left ventricle [46]. Concurrently, cardiac remodelation takes place as a result of increased prevalence of myocardial fibrosis proportional to the extent of obesity and accompanied by chronic inflammation and tissue degeneration. Elevated serum levels of C-reactive protein, TNF-α and IL-6 were observed in obese patients [48]. Another essential factor in the development of the left ventricular hypertrophy is the contribution of neurohumoral mechanisms, as for instance the stimulation of the sympathetic nervous system and the renin-angiotensin-aldosterone system as well as the direct synthesis of aldosterone by adipocytes [49]. AF and its greater incidence in obese patients also increases the risk of HF development via tachycardia-induced cardiomyopathy [50]. Furthermore, cardiac steatosis - the deposition of triglycerides and free fatty acids in the myocardium - both in the interstitium and in the cardiomyocytes, which is positively associated with BMI, contributes to increased mass of the left ventricle. Cardiac steatosis is accompanied by the apoptosis of cardiomyocytes [51].

Therefore it can be stated that, initially, obesity induces a cardiomyopathy characterised by the hypertrophy of the left ventricle, which results in reduced compliance and the development of primarily diastolic dysfunction of the heart with a preserved ejection fraction type of HF (HFpEF) [52]. Over time and with the long-term effects of obesity in the organism, the systolic function of the left ventricle deteriorates and HF with a reduced ejection fraction (HFrEF) develops [53].

### Obesity and treatment of heart failure with reduced ejection fraction

This subgroup of HF is without a question the most widely studied with clearly defined evidence based recommendations for treatment. In general, the algorithm of treatment of symptomatic HFrEF according to the newest 2021 European Society of Cardiology (ESC) guidelines is also recommended for obese patients with HFrEF, i.e. the first step in the pharmacotherapy of symptomatic HFrEF is to initiate treatment with beta blocker, ACE (angiotensin converting enzyme) inhibitor or ARNI (angiotensin receptor-neprilysin inhibitor) and SGLT2i. If still symptomatic and the ejection fraction (EF) is ≤ 35% add a mineralocorticoid receptor antagonist (MRA) and uptitrate to maximum tolerated evidence-based dose; add loop diuretics to relieve symptoms and signs of congestion [54]. If there is further progression of symptoms, several other medications and therapeutic approaches are recommended as listed in the ESC guidelines [54].

In particular, the addition of SGLT2i to the first-line treatment of HFrEF shows substantial progress compared to previous guidelines and reflects the results of the randomized studies with empagliflozin and dapagliflozin in HFrEF patients [55, 56]. Additionally, obese patients can benefit from the updated treatment considering the aforementioned favourable effects of gliflozins on reducing body weight, EAT mass as well as their anti-inflammatory, cardioprotective and renoprotective properties.

There is no conclusive data on the weight reduction in the treatment of HFrEF; however, it was demonstrated that even a slight decrese in body weight was associated with a reduction in the size of the left ventricle, with a decrease in the serum levels of lipids as well as in the levels of the C-reactive protein in obese patients [57]. Considering the fact that the BMI range of 30-34.9 kg/m^2^ is associated with improved short-term as well as long-term survival of obese patients with HF, a number of studies therefore suggest to prefer a low-calorie diet with regular aerobic and anaerobic physical exercise over a rapid weight reduction [44].

### Obesity and treatment of HF with preserved ejection fraction

As mentioned above, the obesity cardiomyopathy leads to primarily diastolic dysfunction, which is reflected in the manifestation of HF in obese patients, who present with HFpEF in the initial stages. Currently, there is limited data regarding the treatment of HFpEF, which is also reflected in the absence of unambiguous recommendations from the ESC for the treatment of HFpEF in comparison to HFrEF. Generally, it is needed to treat underlying diseases (if treatable), and add loop diuretics to relieve signs of congestion. MRA are subject to research, and eplerenone can be considered in selected patients with HFpEF, because it improved the diastolic function of the left ventricle with reduction in its size, along with lowering the risk of rehospitalisations for HF [58]. In addition, the CHARM-Preserved study, which included patients with HFpEF (defined as symptomatic HF and left ventricular EF > 40%) showed that candesartan significantly reduced the number of HF-related hospitalisations in patients with EF > 40%, though no benefit was observed with regards to cardiovascular mortality [56]. SGLT2i hold great expectations as they demonstrated a clear clinical benefit in patients with HFrEF. While several studies are still underway, the results of EMPEROR-Preserved trial have recently been published and showed clinical benefit of empaglifozin in the therapy of HFpEF by decreasing the number of hospitalisations for HFpEF and also reducing the risk of cardiovascular mortality, which was independent of T2D [59]. The result of EMPEROR-Preserved trial together with other ongoing studies in this area, such as DELIVER trial involving dapagliflozin [60] may pave the way for the incorporation of SGLT2i into official recommendations for the treatment of HFpEF and thereby widen the spectrum of therapeutic modalities in this subgroup of HF.

Likewise, the adequate control of other comorbidities such as hypertension is vital. There is information about the so-called obesity paradox even in this group of patients, however clear data about weight reduction is yet to be presented. Nevertheless, weight reduction was associated with improvement in the NYHA class, particularly in the group of patients with an existing HFpEF, who made diet modifications and had increased physical exercise [61].

Finally, glucagon-like peptide 1 receptor agonists (GLP1-RA) seem to be a promising class of drugs for weight reduction in obese individuals, with previously proven cardiovascular protectivity (mostly due to reduction of vascular-related events) [62]. Cardioprotective effect of GLP1-RA in obese individuals was confirmed in the SELECT trial. The trial examined the effect of semaglutide on major adverse cardiovascular events (MACE), in a double-blind, placebo-controlled design. The trial enrolled 17,604 adults with CVD and obesity who were randomized to semaglutide dosed 2.4 mg weekly or placebo. The trial observed a significant 20% reduction in MACE risk for those receiving semaglutide, demonstrating its potential in obesity-associated CVD prevention [63]. In the recently published STEP HFpEF study, treatment with semaglutide (2.4 mg once weekly) in patients with obesity (without T2D) and HFpEF improved HF-related symptoms, physical limitations and ability to exercise, lowered NT-proBNP levels, and resulted in greater weight loss, as compared with placebo. Although the study was underpowered for the assessment of clinical end-points (only 13 HF-related events occurred during study follow-up), there was a trend towards lower incidence of these events in semaglutide-treated patients [64]. The results of this study advocates the need for larger randomized, placebo – controlled studies with GLP1-RA in obese patients with HFpEF to assess (confirm) their ability to reduce clinical HF-related events (such as cardiovascular mortality and HF-related hospitalizations).

### Obesity and treatment of HF with mid-range ejection fraction

Currently, in patients with HF with mid-range ejection fraction (HFmrEF), there is limited amount of clinical data regarding optimal medical treatment of this type of HF [55]. Candesartan actually achieved similar results in this group as in HFrEF considering the time elapsed until the first hospitalisation and repeated hospitalisations. Unlike in HFpEF, candesartan also reduced the cardiovascular risk in this group [56]. Additionally, according to latest ESC recommendations, loop diuretics are recommended in those patients with symptoms and signs of congestion, and ACE inhibitors, beta blockers, MRA and ANRI may be considered in selected patients with HFmrEF to reduce the risk of HF-related hospitalizations and death [54]. However, this recommendation has a level C evidence, and there is no specific study directly examining the optimal pharmacotherapy of HFmrEF in obese individuals, and this issue remains open for future research. In the light of recommendations about weight reduction in obese patients and the established association between weight loss and reduced overall mortality of patients, it is necessary to point out a study of 330 patients with obesity and HFmrEF. In this particular study, a relationship between BMI and mortality of patients with HFmEF was analysed and, surprisingly, individuals with a greater BMI had better survival compared to patients with normal BMI values. The BMI even had a protective effect on survival, although only in the group of patients where HF was not of ischaemic origin [43]. This paradox is explained by the presence of the so-called sarcopenic obesity, therefore lowering the calorie intake is not the only recommended modality in obese patients with HFmrEF but a combination of low calorie diet with aerobic or anaerobic physical exercise should be considered [26].

## Conclusion

Obesity is an independent and modifiable risk factor for AF and HF. In addition, there is an increasing volume of experimental and clinical data that suggests an important role of the EAT in the pathophysiology of AF. Alongside the already well-established pillars of AF treatment, a new, perspective strategy is developing, which aims at lowering the AF risk via lifestyle changes and improvement of cardiorespiratory fitness with the potential of reducing the incidence of AF and thereby alleviating the burden it imposes on the population. Further research is required in terms of weight reduction strategies in patients with AF in the context of cardioembolic stroke, since the data suggests the presence of the obesity paradox here. Studies indicate that obesity initially causes HFpEF via obesity cardiomyopathy and the systolic dysfunction only develops in the later stages. It is also necessary to investigate the effectivity of pharmacotherapy recommended for HFrEF in other types of HF as well due to little available clinical data regarding the treatment of obese patients with HF. Certain progress should be expected by the implementation of SGLTi (based on the latest data from clinical studies in patients with HFrEF and HFpEF); and with novel weight reduction drugs with cardiovascular protectivity, such as GLP1-RA. Undoubtedly, these drugs will be studied with regard to HF-related outcomes in the up-coming days, and may represent a future of obesity-related HF pharmacotherapy. Furthermore, it is very realistic that non-pharmacological treatment of AF and HF will be extensively studied in obese individuals with AF and HFrEF in the near future. Concluding, the issue of optimal pharmacotherapy, non-pharmacological therapy and weight reduction strategy (considering the data regarding obesity paradox in selected patients with HF) in obese patients with HF remains still open for future investigation.

## Data Availability

All the source data are available at the Corresponding Author upon a reasonable request.
